# TRANSLATABLE EXERCISE INTERVENTIONS TO REACH OLDER ADULTS WITH PHYSICAL AND COGNITIVE FRAILTY

**DOI:** 10.1093/geroni/igae098.1476

**Published:** 2024-12-31

**Authors:** Kieran Reid

**Affiliations:** Harvard Medical School, Boston, Massachusetts, United States

## Abstract

Mobility limitations, falls and dementia are leading causes of disability, institutionalization, and mortality among U.S older adults. Considering that the population of adults aged ≥ 65 years is projected to surpass 80 million by 2040, more effective and scalable intervention strategies to maintain mobility, reduce falls or offset cognitive decline are necessary to help older Americans to continue to live independently. This presentation will discuss the rationale, design and implementation of several innovative exercise intervention strategies for older adults with mobility limitations, elevated fall risk, and for older adults at high risk for developing dementia. The session will specifically describe how large-scale clinical trial evidence for the benefits of exercise can be translated to reach more vulnerable older adults in real-world community-based settings. The session will also describe how a digitally delivered multicomponent fall prevention exercise program can be tailored to reach at-risk older adults in their own homes. Overall, it is posited that these innovative interventions hold significant potential as highly scalable approaches for maintaining mobility, preventing falls, and mitigating cognitive decline among rapidly expanding populations of older adults.

